# Diet in Protracted Fevers

**Published:** 1852-01

**Authors:** E. B. Haskins

**Affiliations:** Clarksville, Tenn.


### ARTICLE VI.

*Diet in Protracted Fevers.* A letter to the Editor, by E. B. Haskins, M. D.

Dear Sir: When I saw you last, I promised a contribution to your excellent Journal, and as the continued fevers* of the West are being investigated with increasing interest, I propose, briefly, laying before your readers some suggestions relative to the diet most proper in the protracted stage of these fevers.

On some future occasion I may give my experience in full, in the management of the continued fevers of this locality; but at present will confine my remarks to the single point already mentioned.

It is well known to your readers, who have at all kept pace with the advancement of chemico-physiology, that in the food of man, there are two great classes of alimentary substances, to subserve the wants of the ever-changing organism—the *nitrogenous* and *non-nitrogenous.* The former being destined for the nourishment of the tissues, whilst the latter subserves, mostly, the purpose, by slow combustion, of the generation of animal heat. It is not denied that in the process of disin-

*Usually termed Typhoid Fever, from some semiological likeness to the fever studied and described under that cognomen by Louis, Schomel and Andral of Paris, and subsequently identified in America by Gerhard, Jackson, Pennock, Hale and others, and still more recently in Scotland, Ireland, and England, by Stewart, Kennedy, Jenner and others. It is not difficult to foresee the confusion likely to grow out of the loose application of this name to the continued fevers of this country. The identity of Typhoid Fever is based, essentially, upon *anatomical* peculiarities, and it is to disturb the precision of scientific language to apply this name to our fevers, before the characteristic lesion has been clearly made out. It is not unusual to see a long essay on Typhoid Fever, in every way interesting—except, the author has made *no post mortem dissections of fatal cases*, and, perhaps, some of the most characteristic symptoms of that disease have not been observed by *him!*

tegration, or wasting of the tissues some heat is evolved, and, also, that some fat enters into the composition of those tissues, whilst other portions seem necessary to give symetry to the body and form cushions of support to movable parts ; yet the above classification of alimentary substances, based upon their uses, is regarded mainly as true. It may also be remarked, that that portion of the non-azotized substances, not directly burned off in the circulation is converted into fat** and deposited in the adipose tissue for further use.

This economical disposition of respirable materials, is beautifully illustrated in the habits of hybernating mammalia---that go into their winter retreats loaded with fat, and come out in the spring comparatively lean. As such animals remain physically inactive during the season of hybernation, of course but little waste of muscular or other vital structures takes place--not more than can readily be supplied by the proteinacous compounds already in the vascular system.

Now, when a subject enjoying ordinary health is seized with continued fever, little or no food is required for a number of days ; the blood being charged with azotized matter sufficient to supply the waste from tissual disintegration, whilst in the adipose cells there is already fat enough for repiratory purposes.

But should the disease continue unchecked, the time arises when food becomes imperative---when the limbs become lean and emaciated, and prostration of strength supervenes. All agree as to the *time* for the more prompt and regular administration of food ; but the *kind* of food best suited for this state seems not to have met with so general an agreement. The diet usually prescribed in such cases is animalized waters, as beef tea, chicken water, &c., and that the manner of preparing them is to remove all of the fat as it rises to the surface in the process of boiling. So the patient, it is perceived, ingests nothing but azotized food.

Now what, a priori, will be the course of a protracted fever under such a diatetic system ? It is clear---the adipose tissue being exhausted of its moveable fat, and no starch, gum,

**Dumas, Boussingault and Payen have denied that the animal organism can elaborate fat out of the non-azotized substances; yet the experiments at the Giessen laboratory, under the direction of Liebig, as well as those by Dr. Thompson, of Glasgow, fully sustain the doctrine.

sugar or other combustible material being furnished to the blood, the oxygen of the inspired air seizes upon the tissues, and the brain being, perhaps, the most oxydizable, is the most energetically attacked , and as fatty matter enters largely into its composition,* no adequate reparation can take place---hence delirium, subsultus tendinum, wakefulness, and other manifestation of morbid cerebro-spinal activity†---phenomena too often witnessed at the bedside, and always portends an unfavorable issue.

This hypothetical view of the pathology of the brain, is in some degree, supported by the fact that post-mortem examinations have failed to detect any constant lesion in that organ ; and when congestion or inflammation has been found, it is not violent to presume that it came on as a secondary lesion.

The point in the dietary of protracted fevers, to which I wish to direct attention, is already clear to your mind---that non-nitrogenized substances, as starch, gum, sugar, &c., should be freely administered, instead of the exclusively nitrogenized diet. By thus furnishing respirable materials, the tissues, particularly the *brain*, are protected from the destructive influences of the inspired oxygen. These materials, then, are far more essential to the organism, under such circumstances, than the azotized substances ; since, whilst the muscular system is comparatively at rest, the histological elements undergo very slow disintegration, as is exemplified in hybernating animals.

The non-azotized alimentary substances, as is well known, can be rendered as palatable and inoffensive to the sick, (oils excepted,) as can the nitrogenized. Sweetened gum water, arrow root jelly, barley water, and even the gruel of indian meal are quite palatable and unirritating to the most delicate stomach. To secure, however, all of the possible dietetic wants of the system, they may be alternated with the nitrogenized diet.‡

---

*The adult nervous matter contains about 6 per cent. of *cerebral fat.*

†See Leibig on the effects of starvation in his Animal Chemistry.

‡Dr. Prout was of the opinion that fat should subserve the purpose of tissual nutrition, in general ; though physiologists of the present day, pretty uniformly concur in the opposite opinion.

In conclusion, I will take occasion to remark, that in determining the course of practice upon the theoretical grounds, great caution is necessary, that we commit no extravagances. We should never, from a priori reasoning alone, abandon well established therapeutic usage. The most that may be ventured upon with impunity is the modification and correction of unenlightened experience, and the furnishing materials for such blanks as observation has failed to fill up. Ratiocination is too uncertain a guide to be wholly trusted to in the prosecution of an art like therapeutics, based upon *progressive* science. Kept within these restricted bounds, reason performs her legitimate office in the advancement of a profession that can never be purely empirical or national.

CLARKSVILLE, Tenn. Nov. 5, 1851.

[*Nashville Medical Journal.*

